# Adaptive control of a wheelchair mounted robotic arm with neuromorphically integrated velocity readings and online-learning

**DOI:** 10.3389/fnins.2022.1007736

**Published:** 2022-09-29

**Authors:** Michael Ehrlich, Yuval Zaidel, Patrice L. Weiss, Arie Melamed Yekel, Naomi Gefen, Lazar Supic, Elishai Ezra Tsur

**Affiliations:** ^1^Neuro-Biomorphic Engineering Lab, Open University of Israel, Ra’anana, Israel; ^2^Department of Occupational Therapy, University of Haifa, Haifa, Israel; ^3^The Helmsley Pediatric & Adolescent Rehabilitation Research Center, ALYN Hospital, Jerusalem, Israel; ^4^Accenture Labs, San Francisco, CA, United States

**Keywords:** neuromorphic control, Neural Engineering Framework (NEF), neurorehabilitation, online learning, prescribed error sensitivity, clinical robotic study

## Abstract

Wheelchair-mounted robotic arms support people with upper extremity disabilities with various activities of daily living (ADL). However, the associated cost and the power consumption of responsive and adaptive assistive robotic arms contribute to the fact that such systems are in limited use. Neuromorphic spiking neural networks can be used for a real-time machine learning-driven control of robots, providing an energy efficient framework for adaptive control. In this work, we demonstrate a neuromorphic adaptive control of a wheelchair-mounted robotic arm deployed on Intel’s Loihi chip. Our algorithm design uses neuromorphically represented and integrated velocity readings to derive the arm’s current state. The proposed controller provides the robotic arm with adaptive signals, guiding its motion while accounting for kinematic changes in real-time. We pilot-tested the device with an able-bodied participant to evaluate its accuracy while performing ADL-related trajectories. We further demonstrated the capacity of the controller to compensate for unexpected inertia-generating payloads using online learning. Videotaped recordings of ADL tasks performed by the robot were viewed by caregivers; data summarizing their feedback on the user experience and the potential benefit of the system is reported.

## Introduction

Over the past few decades, robotic arms have been demonstrated to be immensely valuable for a broad spectrum of applications, ranging from space debris mitigation (Nishida et al., 2009) and the exploration of celestial bodies (Brooks et al., 2022) to fruit harvesting (Font et al., 2014) and robot-assisted surgeries (Naziri et al., 2019). The development of assistive smart robots was initiated four decades ago (Udupa et al., 2021). Since then, it has been established as one of the essential frontiers in neurorehabilitation, enhancing the sense of independence and well-being in people with disabilities. Assistive robots such as robotic walkers, exoskeletons (wearable robots), prostheses, powered wheelchairs, and wheelchair-mounted robotic arms, provide structure, support, and energy to enable independent function and activities of daily living (ADL) by people with physical disabilities (Argall, 2018). Particularly, wheelchair-mounted robotic arms were shown to support people with upper extremity disabilities with various ADL such as picking an object from a shelf or holding a cup, increasing the users’ sense of independence (Maheu et al., 2011). However, the associated cost of assistive robotic arms contributes to the fact that such systems are not commonly found. Furthermore, as wheelchair-mounted robotic arms feed on the chair’s battery, power efficiency can become an important concern. Therefore, the development of a wheelchair-mounted robotic arm, with an energy efficient adaptive control, can become an important step forward in rehabilitation robotics.

A conventional robot controller, such as a proportional, integral, derivative (PID) controller, applies correction signals based on the system’s error’s proportional, integral, and derivative terms (Ang et al., 2005). PID integrates three error modalities to provide the desired actuation, such that the system will approach a target position. While conventional—PID driven–computational motion planning has been shown to handle intricate maneuvers in challenging convoluted environments, when compared with biological control, they fall short in terms of energy efficiency, robustness, versatility, and adaptivity to changing conditions (DeWolf et al., 2016; DeWolf, 2021; Volinski et al., 2022).

One of the ways biological motor control circuits efficiently handle stochastic conditions is by efficiently implementing an adaptive control scheme. Adaptive motor control is long known to be mediated by projection neurons involving the basal ganglia and the neocortex, providing vision and proprioception-driven real-time error-correcting adaptive signals with which a dynamic motor control could be efficiently realized (Graybiel et al., 1994). Failure to generate these error-correcting signals can manifest as Parkinson’s (Burget et al., 2015) or Huntington’s (Smith et al., 2000) brain disorders. While adaptive control could be implemented in conventional neural computational frameworks (Cong and Liang, 2009), spiking neuronal architectures were shown to provide increased performance with lower energy consumption (DeWolf et al., 2020). A typical spiking neural network (SNN) comprises densely connected, spike-generating neuron weighted fabric through which spikes are propagated, thus closely emulating biological neural networks (Tsur, 2021). SNNs were recently used to neuromorphically implement PID (Rasmus et al., 2020; Zaidel et al., 2021).

Neuromorphic control algorithms acquire some of the advantages of biological motor control. They have been shown to outperform PID-based implementation of the required nonlinear adaptation, particularly in handling high DoF systems (DeWolf, 2021). Neuromorphic adaptive control utilizes online learning with spiking neural networks to account for unexpected environment perturbations. For example, neuromorphic adaptation was recently implemented using an adaptive version of the spike-timing-dependent plasticity (STDP) learning rule, demonstrating adaptation with state-of-the-art power consumption (Gautam and Kohno, 2021). Adaptive robotic control was previously demonstrated in various settings. For example, a neuromorphic vision-based adaptive controller was recently designed to control an unmanned aerial vehicle moving at high speed (Vitale et al., 2021). The authors propose a neuromorphic controller with event-based visual feedback computed on a neuromorphic chip (Loihi). This control system was shown to outperform the state-of-the-art high-speed event-driven controller.

A prominent method for the design and neuromorphic systems is the Neural Engineering Framework (NEF) (Eliasmith and Anderson, 2003). NEF is a theoretical framework, which was implemented as Nengo, a “neural compiler,” allowing the translation of high-level neural descriptions to functional large-scale SNNs (Bekolay et al., 2014). NEF was utilized to design a wide range of SNN-driven applications, ranging from robotic control (Zaidel et al., 2021) and visual processing (Yun and Wong, 2021) to perception (Eliasmith and Stewart, 2012) and pattern recognition (Wang et al., 2017). NEF was shown to be incredibly versatile, as a version of it was compiled to work on both analog and digital neuromorphic circuitry (Voelker, 2015; Hazan and Tsur, 2021). Power comparison between neuromorphic NEF-driven implementation of adaptive control to conventional CPU and GPU-based implementation, demonstrated increased power efficiency while preserving similar latency performance (DeWolf et al., 2020).

In this work, we demonstrate a neuromorphic adaptive control of a wheelchair-mounted robotic arm deployed on Intel’s Loihi chip. We used proprioceptive feedback provided by an affordable accelerometer in conjunction with a neuromorphic integrator to continuously provide the system with the robot’s current state in real-time. Similar to biological adaptive control, these readings are used to provide the controller with motion guidance and adaptive signals, allowing it to account for kinematic changes in real-time.

## Materials and methods

**FIGURE 1:**
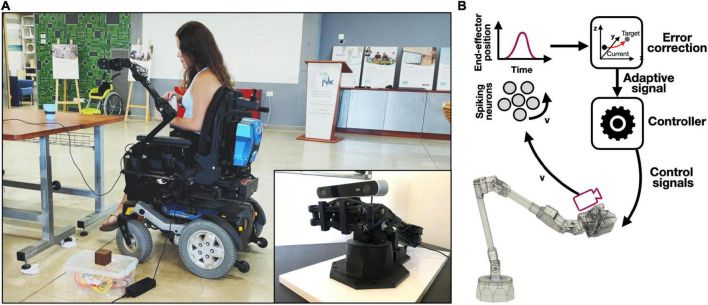
Acceleration-mediated adaptive control of a wheelchair mounted robotic arm. **(A)** The complete system where the accelerator is embedded within the stereo camera, which is mounter on the arm’s end-effector; demonstrated by Yuval Zaidel (author; published with permission); **(B)** control framework schematic: accelerometer-driven velocity readings are neuromorphically integrated with spiking neurons, allowing the derivation of the arm’s position. The arm’s position is compared to its desired state providing error correcting adaptive signals. Adaptive signals are introduced to the controller for accurate final positioning.

In this work, we propose a NEF-driven SNN, deployed on Intel’s Loihi neuromorphic chip for adaptive control of a wheelchair-mounted robotic arm (Figure 1A). We used accelerometer-generated velocity readings as feedback, feeding them into a neuromorphic integrator to continuously provide the system with the robot’s current state in real-time (Figure 1B). Continuous state estimation allows the system to adaptively control the trajectory of a robotic arm. Our NEF-defined SNN-driven integrator is a dynamical system with which incoming velocity readings are integrated to monitor the system’s state (position). The robotic system, the Loihi board, and the NEF are described in sections “Robotic system,” “Neuromorphic hardware,” and “The neural engineering framework,” respectively. The neuromorphic integration for state estimation is described in section “Neuromorphic integration for state estimation.” Position estimation was used by the controller to guide the arm’s trajectory using online learning. Underlying the proposed online learning is the prescribed error sensitivity (PES) learning rule. PES is a biologically plausible supervised learning rule that modifies a connection’s weight in a SNN such that an error signal is minimized (Voelker, 2015). This neuromorphic online learning-driven control scheme allows the robotic system to continuously generate adaptive signals during motion, using them to correct its posture as it reaches its targets efficiently. PES is described in section “Prescribed error sensitivity.” We used the system to adaptively respond to kinematic changes (lifting heavy objects). The adaptive control is described in section “Adaptive control.” The arm trajectories were designed to reach three key target points, which were shown to be important to ADL tasks (Routhier et al., 2014; Beaudoin et al., 2018): lifting an object from higher and lower grounds as well as serving a user with a cup of water.

### Robotic system

In this work, we used a six degrees of freedom robotic arm comprised of nine servo actuators (seven Dynamixel’s XM540-W270 actuators and two Dynamixel’s XM430-W350 actuators; two sets of two actuators were assigned to modulate two joints to increase capacity load). The XM540 actuators were used to actuate the arm’s joints and are characterized by a stall torque of 10.6 Nm (at 12 v input). The XM430 actuators were used to manipulate the end-effector (grasping) and are characterized by a stall torque of 4.1 Nm (at 12 v input). Each actuator can handle a 40 N radial load and features a Cortex-M3 embedded controller. To retrieve the joint’s current state (angular rotation), each actuator was coupled with a contactless 12-bit absolute encoder. Actuators were manufactured by ROBOTIS. The arm chassis was assembled from ridged and lightweight T-slot extruded aluminum rods, aluminum brackets, industrial-grade slewing bearings, and a 3D-printed gripper by Interbotix. Control was deployed on Intel’s Loihi neuromorphic chip and communicated to the robotic system using a TTL half-duplex asynchronous serial communication, handled by Dynamixel’s U2D2 control board. Overall, the arm design provides an 82 cm reach, a 1.64 m span, 1 mm accuracy, and a 750-gr payload. We mounted a Stereo Labs’ ZED stereo camera on the arm’s end-effector and used its embedded accelerometer for velocity readings. Manual control was established through a Bluetooth-connected PlayStation’s DualShock4 controller. The robotic arm was mounted on an electric wheelchair by the technical team of ALYNnovation, the innovation center of ALYN hospital.

### Neuromorphic hardware

Neuromorphic control was implemented in Nengo (Bekolay et al., 2014) and deployed on Intel’s neuromorphic research chip Loihi (Davies et al., 2018) using the nengo_loihi library (Lin et al., 2018). The nengo_loihi library provides an API for both an emulator and a Loihi-specific Intel’s NxSDK-based compiler, allowing model deployment on the board itself. We used Intel’s Kapoho Bay, a USB-based neuromorphic processor, which incorporates 2 Loihi chips. Each Loihi chip features x86 cores (for spike routing and monitoring) and 128 neuron-cores, each supporting 1,024 neurons. The Kapoho Bay has overall 256 neuromorphic cores with 262,144 neurons and 260,000,000 synapses.

### The neural engineering framework

The Neural Engineering Framework is a theoretical framework for neuromorphic encoding, decoding, and transforming high-dimensional mathematical constructs with ensembles of spiking neurons (Eliasmith and Anderson, 2003). With NEF, high-level descriptions of functional neural circuits can be translated down to the level of the interconnected weighted fabric of spiking neurons. With NEF, an ensemble of neurons distributively encode mathematical constructs, where each neuron is characterized by a response dynamic (tuning curve). A spike train δ_*i*_ of neuron *i* in response to a stimulus *x* is defined as:


(1) $$\delta_{i}\,\left(x\right)=G_{i}\,\left[\alpha_{i}\,e_{i}+J_{i}^{b}\right],$$

where *G*_*i*_ is a spiking neuron model (e.g., leaky integrate and fire), α_*i*_ is a gain factor, *e*_*i*_ is the neuron’s encoding vector (preferred stimulus), and $J_{i}^{b}$ is a background current. The encoded stimulus *x* can be linearly decoded as $\hat{x}$using:


(2) $$\hat{x}=\sum_{i}^{N}a_{i}\,\left(x\right)\,d_{i},$$

where *N* is the number of neurons, *d*_*i*_ is a linear decoder that was optimized to reconstruct *x* using least-squares optimization, and *a*_*i*_(*x*) is the postsynaptic response of neuron *i* to *x* defined as:


(3) $$a_{i}\,\left(\text{x}\right)=\sum h_{i}*\delta_{i}\,\left(t-t_{j}\,\left(\text{x}\right)\right),$$

where h_*i*_ is the synaptic response function (an exponential function, inspired by the neurotransmitter-dynamic at the synapse), δ_*i*_(*t* − *t*_*j*_(x)) is the spike train produced by neuron *i* in response to stimulus x with spike times indexed by *j*, and * refers to mathematical convolution.

Spikes propagate from one ensemble to another through weighted synaptic connections (decoding weights d*^f^*) realizing a mathematical transformation *f*(*x*). The decoders d*^f^* can be optimized to define a desired *f*(*x*) and formulated as a weight matrix *w*_*ij*_(*x*):


(4) $$w_{i\,j}=d_{i}⊗e_{j},$$

where ⊗ is the outer product operation; *i* is the neuron index in spike source ensemble *A* and *d*_*i*_ are the corresponding decoders; *j* is the neuron index in the target ensemble *B* and *e*_*j*_ are the correspocnding encoders. *d*_*i*_ and *e*_*j*_ are optimized to transform *x* (neuromorphically represented by *A*) to *f*(*x*) (neuromorphically represented by *B*).

The noise characteristics of NEF-based representation is based on the decoder-induced static noise, and it is proportional to 1/*N*^2^ where N is the number of neurons. Synaptic time constants also constrain neuromorphic implementations. Reducing these time constants inhibits the integration dynamic. A detailed description of neuromorphic integration with NEF is given in (Tsur, 2021).

### Neuromorphic integration for state estimation

Dynamic behavior can be realized by combining NEF’s neuromorphic representation and the transformation of numerical entities through the recurrent connection of neuronal ensembles. NEF can therefore be used to resolve the general dynamic form:


(5) $$\frac{\partial x}{\partial t}=A\,x\,\left(t\right)+B\,u\,\left(t\right)$$

where *u*(*t*) is input from some neural ensemble, *A* and *B* can be resolved from *A*′ = τ*A* + *I* and *B*′ = τB, respectively, where A′ is the recurred connection, B′ is the input scaling factor, *I* is the identity vector, and τ is the synapse’s time constant (Tsur and Rivlin-Etzion, 2020). Here, we used this dynamical system to implement a neuromorphic integrator, where velocity measurements are integrated to monitor the arm’s end-effector position. A neuromorphic integrator can use a velocity input signal *v* to derive a position *x* using *x* = ∫*v*, or by solving $\frac{\partial x}{\partial t}=v$. In terms of Eq. 5, here, *A* = 0 and *B* = 1, resulting in A′ = τ ⋅ 0+I = 1 (a simple recurrent connection) and B′ = τ ⋅ 1 = τ (multiplying the velocity readings by τ).

### Prescribed error sensitivity

Neuromorphic transformation is governed by synaptic weights, which connect one neuron ensemble to another. While these weights can be calculated in build-time, they can also be modulated or learned in real-time. Real-time learning is of particular interest in various areas of machine learning and robotics, as it allows the incorporation of unknown environmental perturbations in the robot’s motion planning. Real-time learning can be implemented with NEF using the PES learning rule, a biologically plausible supervised learning rule, which strives to modulate connections’ decoders *d* to minimize an error signal *e*. Here, the error signal is calculated as the difference between the robot’s desired position *x* and its approximated representation $\hat{x}$, while applying weight update △*d* with the rule:


(6) △⁢d=λ⁢e⁢δ

where λ is the learning rate, and δ is the neuron’s spiking rate. Note that *e* goes to 0 exponentially with rate γ, when *a*−λ||δ||^2^ (denoted γ) is larger than –1. PES is described at length in (Voelker, 2015).

### Adaptive control

Our torque *u* –based robot control is governed by:


(7) $$u=J^{T}\,M_{x}\,u_{x}-K_{v}\,M\,\dot{q}$$

where *J*^*T*^ is the Jacobian matrix, which approximates the relationship between control forces in task space and actuation in joint space in real-time (“Jacobian on-the-fly”); *M*_*x*_ is the inertia matrix (in task space) with which the controller accounts for the inertia generated by the arm’s own movement; *u*_*x*_ is the force (torque) vector in task space; and $K_{v}\,M\,\dot{q}$ is a velocity error term calculated by estimating the lifted body’s inertia. *u* is calculated iteratively, as the value of *u*_*x*_ is recalculated along with the arm trajectory as the arm’s end-effector gets closer to its target by comparing the desired position and the arm’s current position, as determined by our neuromorphic velocity integrator. This iterative calculation is halted when the arm is within some accuracy threshold or when *u*_*x*_ is small enough (here, 0.5 mm).

DeWolf et al. (2016) proposed a NEF-driven adaptive control algorithm, which they named the recurrent error-driven adaptive control hierarchy (REACH) model. REACH is powered by PES and open-sourced by Applied Brain Research Inc. The model has been demonstrated to control a planar three-link, nonlinear arm through intricate trajectories. REACH can support adaptive control, efficiently responding to environmental changes, such as a sudden force field (e.g., lifting a cup full of water instead of an empty one) or changes in the mechanical characteristics of the robotic arm (e.g., joints’ tear).

REACH adaptive control is governed by:


(8) $$u=J^{T}\,M_{x}\,u_{x}-K_{v}\,M\,\dot{q}+u_{a\,d\,a\,p\,t}$$

where *u*_*adapt*_ is the adaptive error correction signal. *u*_*adapt*_ is calculated using PES, as described above. We used PES to estimate *u*_*adapt*_ by comparing the desired position and the arm’s current position, as determined by our neuromorphic velocity integrator. A full description of REACH is available in DeWolf et al. (2016).

## Results

### Motion guidance

**FIGURE 2:**
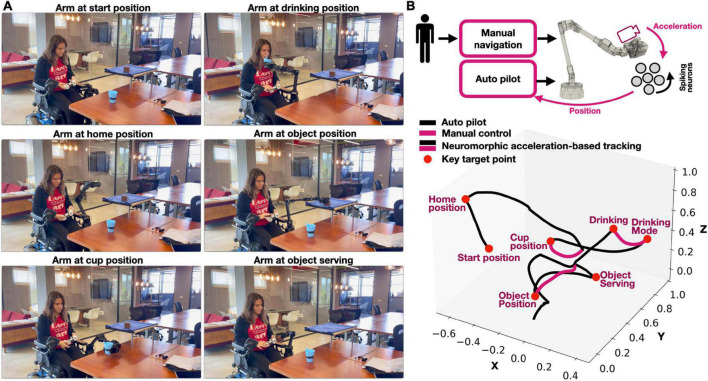
**(A)** Screenshots from the various stages of the robotic assisted activities of daily living (ADL) demonstration. A video is available as a Supplementary Information; demonstrated by Yuval Zaidel (author; published with permission); **(B)** motion guidance of a wheelchair mountet robotic arm using both manual control and automatic motion guidance (auto pilot) to reach several ADL-related key points.

We defined a few ADL-related key target points in space, through which we guided the robotic arm’s trajectory. Motion guidance was divided into two parts. In the first part, motion is automatic, and the arm is actuated toward a predefined target point (e.g., table). In the second part, to accurately approach an object (e.g., a cup on the table), the arm is manually controlled by the user using a wireless remote controller (Figure 2). We defined two ADL tasks: drinking from a cup and “pick and serve.” We initiate the robot at its resting mode for both scenarios, raising it to a home position afterward (Figure 1). In the drinking scenario, the arm approaches the table automatically, with its end effector oriented in a cup-holding position. The user uses his remote control to carefully approach the cup with the arm, finally activating the gripper to hold it. Once gripped, the arm automatically positions itself by the head of the user in a drinking-oriented posture. The user can now control the arm manually, getting the cup closer to his mouth. Once done, the controller automatically returns the cup back on the table. In the pick and serve task, the arm automatically positions itself by the high shelf in a gripping-oriented posture. The user manually controls the arm to approach and grip the object. Once grasped, the arm serves the object to the user’s hands. The arm can then either return the object back on the shelf or go back to its home position.

The arm’s trajectory during both tasks is shown in Figure 2. Both manual and automatic motion guidance (autopilot) controls are fed with the neuromorphically derived robot position (Figure 2, top right). The arm trajectory shown in Figure 2 demonstrates an accurate transversal through each of the target points. A video with an overlayed raster plot (demonstrating spiking activity) and annotated stages is available as a Supplementary Video 1.

**FIGURE 3:**
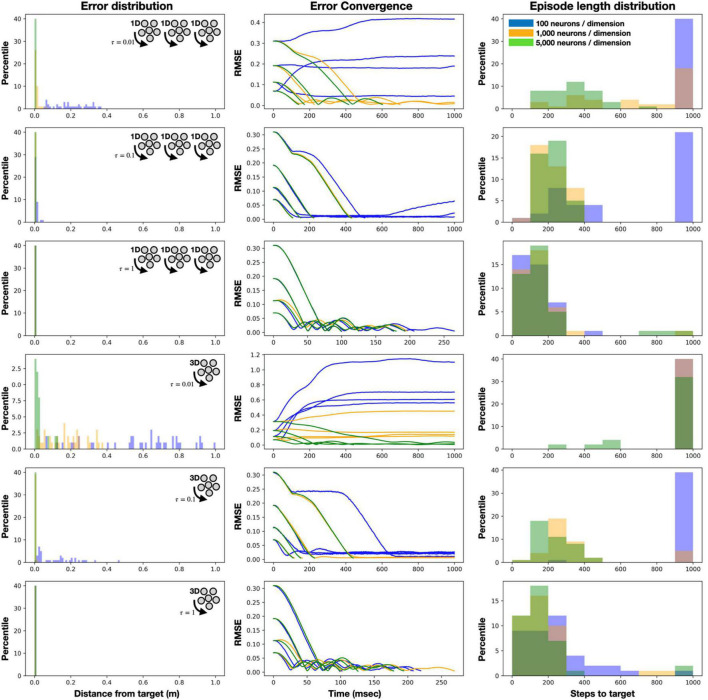
Control evaluation [error distribution and convergence, and episode length (steps to target)] with a neuromorphic integrator featuring 100, 1,000, and 5,000 neurons per dimension, three unidimensional integrators or one 3D integrator and three synaptic constants: 0.01, 0.1 and 1 s. Results were obtained from reaching 100 randomly positioned target points.

We further evaluated our system in various configurations by measuring error’s (distance from target) convergence and distribution, as well as the number of steps required to reach a target. Results were obtained from 100 randomly positioned target points. We evaluated two neural architectures to track the arm’s position in each of its three axes (*x*, *y*, and *z*): (1) three unidimensional neuromorphic integrators and (2) one 3D integrator. In both cases, neurons’ tunning curves were randomly distributed. Each case was also evaluated with a different number of neurons per dimension (100, 1,000, and 5,000) and with various values of synaptic constants (tau = 0.01, 0.1, and 1 s). Results are shown in Figure 3. As expected, when compared to a single high-dimensional ensemble, using three unidimensional neuromorphic integrators is preferable as they can more efficiently span the representation space (assuming these dimensions are independent). Neurons’ tuning curves are mainly governed by their intercepts—the input value from which they respond with an increased firing rate—which defines the neurons’ representational capacity, especially in higher dimensions. For example, while a unidimensional neuron with a 0.75 intersect (inputs are normalized between −1 and 1) will fire spikes for 25% of the represented space, in 2D, this neuron would fire for only 7.2% of that space. In higher dimensions, the proportions become exponentially smaller, resulting in many neurons which are either always active or completely silent, thus, providing a poor representation (Zaidel et al., 2021). In our case, we show that with fast synaptic constants (τ < 0.1*ms*) and a small number of neurons (*N* < 1,000), the noisy integration results in a highly distributed error and slow to non-converging error. When a larger number of neurons are allocated for representation, errors rapidly converged to zero (∼150 ms), the required steps toward the targets diminished, and the error distribution is remarkably low (Figure 3). These results demonstrate the controller’s robustness regarding noise. Noise was introduced here to the system as a product of the neuromorphic representation error, which as was described above, is inherited to neural representation in general and, in particular, to NEF. The results outline the required neuronal resources (number of neurons) needed to handle the introduced noise in different control designs (1D/3D neuron ensembles).

### Adaptive control

**FIGURE 4:**
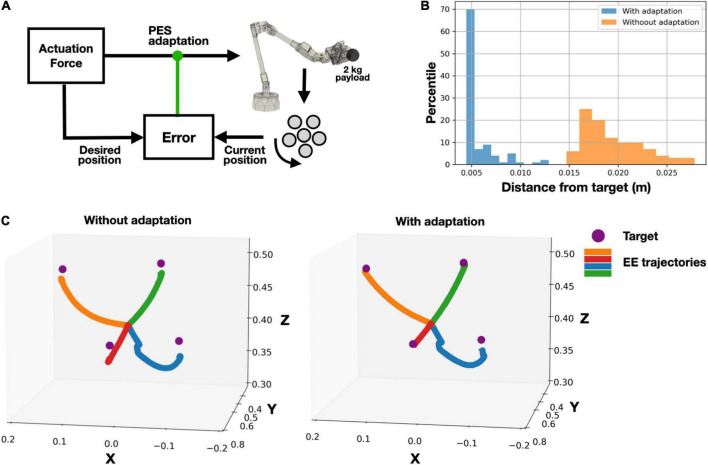
Adaptively controlled robotic arm, while manipulating a 2 kg payload, using acceleration-derived positioning feedback. **(A)** System’s schematic: **(B)** error (distance from target) distribution, evaluated on 100 target points; **(C)** example of reaching four target points with and without adaptive control.

We further evaluated our model with a PES-governed adaptive control while manipulating a 2 kg payload (Figure 4). We assessed the performance of both our naïve (Eq. 8) and adaptive controller (Eq. 9) while reaching 100 randomly positioned target points. Aided by acceleration-derived positioning feedback, the adaptive controller outperformed the naïve version, obtaining closer to the target point final positioning (Figure 4B). We further illustrate reaching four target points, with and without the adaptive signals, demonstrating a superior final positioning while maintaining similar trajectories (Figure 4C).

### Participatory design

Despite the widespread agreement regarding the potential benefits of robotic rehabilitation, the designed technologies, do not often match the requirements of patients and caregivers. This greatly impedes their adoption in healthcare (Arnrich et al., 2010). A design approach that is driven by a “user-centered” or “participatory design” viewpoint aims to counter this limitation by identifying and incorporating the requirements and end users’ needs at each stage of the design process (Bergold and Thomas, 2012; Sivan et al., 2014; Eslami et al., 2018). Questionnaires are among the tools that have been developed to identify end-user requirements (Shah et al., 2009). Here, we obtained design feedback from 10 care stakeholders (occupational therapists, technology developers and distributors, researchers, and administrators) who had 2–30 years of experience with a wide range of assistive technologies (powered wheelchairs, computer-based access, and communication devices). They rated their views on key user experience questions that were posed via an online Google form questionnaire. The exposure of 9 out of 10 of the participants to robotic arms included using one with patients, seeing demonstrations of its use, or reading reports in the literature.

They viewed two video clips demonstrating the robot arm performing the same ADL tasks that have been described above. Eighty percent of the responders agreed or strongly agreed that its appearance is acceptable and that it is safe for use; no one thought that it was not acceptable or not safe for use. The most important issues for safe use of a robotic arm were reported to be limiting its speed of movement (especially when near the user’s “personal space”), easy access to a “panic” button (to stop the robot immediately) and limiting the force that it can exert. All respondents thought that a robot arm should cost less than $2,500 with 90% preferring it to cost less than $1,000. The respondents were asked to report the tasks that they consider to be most important to be performed by a robotic arm. All considered eating or drinking and work or educational tasks (e.g., access to a computer) to be important or very important. All but one respondent considered reaching for items, self-care, and communication (e.g., access to alternative communication devices) to be important or very important. While not being a statistically validated survey, this feedback showcase the importance of having affordable robotic arms for wheelchair users.

## Discussion

Biological motor control is uniquely responsive and adaptive, allowing organisms to quickly respond to environmental changes. Biological control uses visual and proprioception cues to evaluate the body’s current state, dynamically modulating motor commands, such that environmental changes could be efficiently compensated. While a conventional PID controller could consider kinematic changes in the system, such as object manipulation of an unknown dimension or weight, to accommodate new motions and surroundings, it might entail extensive re-tuning of the control parameters. Adaptive spiking neural networks were demonstrated to handle such tasks with remarkable efficiency (DeWolf, 2021).

NEF-driven adaptive control was previously extensively evaluated. For example, DeWolf et al. (2020) compared the power consumption of neuromorphic NEF-driven implementation of adaptive control to conventional CPU and GPU-based implementations. They found 4.6× and 43.2× improved power consumption for execution over CPU and GPU, respectively, while preserving similar latency performance (PD: 2.91 ms, PID: 2.95 ms, adaptive on neuromorphic hardware: 3.08 ms, adaptive on CPU: 3.13 ms, and adaptive on GPU: 4.38 ms). In that research, however, the authors used expensive vision-based proprioception. In this work, we used proprioceptive feedback provided by an affordable accelerometer in conjunction with a neuromorphic integrator to continuously provide the system with the robot’s current state in real-time. We further analyzed the performance with various neuromorphic configurations and evaluated it in a real-life case study. This capacity of smart motor control is particularly interesting in a human collaborative-assistive setting. As was shown in our participatory clinical survey, particularly for wheelchair users, the system cost is a critical factor, contributing to the fact that wheelchair-mounted robotic arms are not commonly found. We demonstrate that neuromorphic implementations of adaptive control may allow the design of less expensive assistive robotic arms, allowing them to exhibit high performance with relatively inexpensive parts and high energy efficiency.

We show that by using a neuromorphic integrator to monitor the state, or the position, of a wheelchair-mounted robotic arm, the generated feedback to a controller can (1) guide the arm’s trajectory and (2) provide adaptive error-correcting signals when environmental conditions (e.g., payload). We evaluate the system by (1) addressing ADL-related arm trajectories and (2) reaching hundreds of randomly positioned target points. Our analysis shows that synaptic constants, the number of neurons, and the neuronal architecture dramatically constrain the controller performance. We show that low dimensional representation (1D), long synaptic constants (τ < 0.1*ms*), and sufficient neural resources (*N* > 1,000) are required to provide robustness and fast convergence (∼150 ms; sub 200 ms is required to avoid a latency bottleneck, considering the robotic system response time).

Furthermore, we show that adaptive control is essential for accurate navigation as reaching the desired target point would require compensating for the payload-generated excessive inertia forces. We show that while a naïve control model which did not take payload into account could not reache its target destinations, an adaptive controller which considers feedback from neuromorphic positioning integrators could compensate for this new environmental condition. Our adaptive controller dynamically generated adaptive signals with which the arm could modulate its dynamics, allowing it to accurately reach its destinations. This capacity of smart motor control is particularly interesting in human collaborative-assistive settings.

To conclude, we provide neuromorphic design guidelines for such an adaptive controller and demonstrate its computational capacity. We plan to continue incorporating an iterative participatory design approach to test the robot with additional users and with further constraints.

## Data availability statement

Publicly available datasets were analyzed in this study. This data can be found here: https://github.com/NBELab/Adaptive_arm_control.

## Ethics statement

All experimental procedures were approved by the Ethical and Guidelines Committee of ALYN Hospital, supervised by the Israeli authorities, and conformed to the National Institutes of Health (NIH) guidelines. Written informed consent was obtained from all participants for their participation in this study.

## Author contributions

EE conceptualized and supervised the research. EE, YZ, and ME wrote the code and performed the experiments. NG, AM, and PW conducted and analyzed the participatory design aspects of the study. All authors were involved in research design and manuscript preparation.

## Supplementary material

Supplementary Video 1 A demonstration of a neuromorphic motion guidance of a wheelchair mounted robotic arm. Demonstrated by Yuval Zaidel and published with permission. The full video in a higher resolution is available at https://youtu.be/BcHqGKHkq3g.
